# Coordinators in the return-to-work process: Mapping their work models

**DOI:** 10.1371/journal.pone.0290021

**Published:** 2023-08-10

**Authors:** Veronica Svärd, Erik Berglund, Elisabeth Björk Brämberg, Niklas Gustafsson, Monika Engblom, Emilie Friberg

**Affiliations:** 1 Unit of Social Work, Department of Social Sciences, Södertörn University, Huddinge, Sweden; 2 Division of Insurance Medicine, Department of Clinical Neuroscience, Karolinska Institutet, Stockholm, Sweden; 3 Medical Unit Social Work in Health, Karolinska University Hospital, Stockholm, Sweden; 4 Department of Public Health and Caring Sciences, Uppsala University, Uppsala, Sweden; 5 Unit of Intervention and Implementation Research for Worker Health, Institute of Environmental Medicine, Karolinska Institutet, Stockholm, Sweden; The John Paul II Catholic University of Lublin, POLAND

## Abstract

**Purpose:**

In recent decades, many countries have implemented return-to-work coordinators to combat high rates of sickness absence and insufficient collaboration in the return-to-work process. The coordinators should improve communication and collaboration between stakeholders in the return-to-work process for people on sickness absence. How they perform their daily work remains unexplored, and we know little about to what extent they collaborate and perform other work tasks to support people on sickness absence. This study examines which work models return-to-work coordinators use in primary healthcare, psychiatry and orthopaedics in Sweden.

**Methods:**

A questionnaire was sent to all 82 coordinators in one region (89% response rate) with questions about the selection of patients, individual patient support, healthcare collaboration, and external collaboration. Random forest classification analysis was used to identify the models.

**Results:**

Three work models were identified. In model A, coordinators were more likely to select certain groups of patients, spend more time in telephone than in face-to-face meetings, and collaborate fairly much. In Model B there was less patient selection and much collaboration and face-to-face meetings. Model C involved little patient selection, much telephone contact and very little collaboration. Model A was more common in primary healthcare, model C in orthopaedics, while model B was distributed equally between primary healthcare and psychiatry.

**Conclusion:**

The work models correspond differently to the coordinator’s assignments of supporting patients and collaborating with healthcare and other stakeholders. The differences lie in how much they actively select patients, how much they collaborate, and with whom. Their different distribution across clinical contexts indicates that organisational demands influence how work models evolve in practice.

## Introduction

The individual and social costs of sickness absence (SA) are a great challenge for most societies. One challenge to reducing SA rates is insufficient communication and collaboration between stakeholders [1, 2]. To ameliorate this, many countries have implemented return-to-work (RTW) coordinators with the task of improving communication and collaboration between various stakeholders involved in the RTW process, for example, the healthcare staff, employer, employment services, Social Insurance Agency (SIA), and the social services. Several literature reviews [3–5] and a meta-analysis of randomised controlled trials [6] have found moderate evidence of RTW coordination resulting in a higher likelihood of RTW for people on SA; other reviews [7, 8] have found no effect. The variation in results is due to such factors as differing patient populations and differing inclusion criteria for different types of study. A scoping review of the impact and role of RTW coordinators for people with mental ill-health [8], concluded that coordinating interventions can be time consuming, and the authors called for studies that clearly define the role, work strategies and actions of RTW coordinators, because these factors determine whether the RTW coordinator has an impact on the SA and RTW rates. Without more in-depth information about such actions, it can be hard to guide RTW coordinators on how to perform their work so their actions will have an impact on the SA and RTW rates.

Existing research into the role of RTW coordinators often describes a range of possible work tasks, although an exhaustive job description is often missing. Some of the tasks mentioned are: having regular contact with the person on SA [9–11], providing ergonomic and workplace assessments [9], identifying barriers to RTW [9, 10], designing and following up a RTW plan [9, 12, 13], and providing workplace conflict resolution and social problem-solving [10, 13, 14]. Coordinators also interpret laws, policies and regulations applying to persons on SA and their RTW [9], and give information and advice about diagnosis, rehabilitation measures, the sickness insurance system and social support [10, 11], as well as reassuring and supporting the patient [9, 11, 12]. Other important duties are facilitating communication and agreement between the stakeholders [10, 11, 13–16] and collaborating with them [9–12, 17, 18]. This wide range makes it difficult to identify exactly which specific components are most important for a successful RTW process [3]. One exception is a recent systematic review that found strong evidence that interventions where RTW coordinators having face-to-face contact with people on SA increase their RTW rates [19]. Several literature reviews conclude, however, that much research into RTW coordination lacks a detailed description of the context, the actual nature of the interventions [6], and the extent to which different stakeholders are involved [7]. This underscores the importance of gaining more in-depth knowledge about, for example, which specific work tasks are actually carried out and how often. Without such information we cannot answer the question as to why a particular intervention does or does not have an effect on outcomes [20] such as improving work ability and reducing SA.

The RTW coordinators’ role and work tasks can differ internationally according to the particular welfare or social insurance system, the setting, and the types of people on SA. In Sweden, all workers with income from work, unemployment or parental benefits can be granted full- or part-time SA benefits up to 80 percent of lost income from the SIA if having reduced work capacity due to disease or injury, irrespective of this being related to their workplace. Whereas in many countries RTW coordinators are often employed by insurance companies or workplace organisations, in Sweden they usually work for the public health services, most frequently employed in primary healthcare (67.5%), psychiatry (11.6%) and orthopaedics (4.4%) [21]. These three healthcare settings have been outlined as dealing with more SA cases compared to other settings, whereby RTW coordinators, also called rehabilitation coordinators in Sweden, have been successively introduced here over the past couple of decades. To ensure equality in access to RTW coordination, health services have been obliged, since 2020, to offer all patients on SA coordination of their rehabilitation process if needed [22]. The RTW coordinators therefore work with different patient populations in these settings, with the most frequent being common mental disorders and musculoskeletal disorders in primary healthcare, psychiatric disorders in psychiatry, and orthopaedic injuries in orthopaedic clinics. Irrespective of their different patient populations and healthcare settings, the RTW coordinators have been imposed three main assignments: giving individual support to the person on SA, collaborating within the health services, and external collaboration with other stakeholders involved in the RTW process [22]. The role is outlined in a handbook for RTW coordination [23] which describes a range of possible or recommended tasks. However, the implementation of the RTW coordinating function was not followed by any recommendations about how the work should be carried out and there are no suggested evidence-based methods. Many decisions are consequently left to the individual RTW coordinator. One previous interview study [15] of Swedish RTW coordinators confirms that they lack guidance in how to carry out the job on a daily basis, leading to a variation in practice and the risk of drift from the original assignments. There appear to be differences in how coordinators perform their work and confusion about best practice and the type of patient they should work with. For example, in contrast to the assignment to offer patients RTW coordination based on individual assessments of their needs, some qualitative studies outline that coordinators in primary healthcare tend to offer their support selectively to those who are, e.g., younger than 40 years old, have less complicated situations, or have a workplace to return to, and refuse to take on people who have exceeded 180 SA days [24, 25]. If, and how, RTW coordinators select some groups of patients, and refuse others, is therefore a question of how they choose to carry out their work, which can have direct consequences for the patient’s possibilities to obtain qualified RTW coordination and support.

The aim of this study was to explore which work models the RTW coordinators use in practice in three clinical areas: primary healthcare, psychiatry and orthopaedics. With the objective of mapping and describing the different work models, we explored how often coordinators carried out certain tasks, how much time they spent on them, how much they collaborate with stakeholders, and whether they select certain groups of patients.

### Defining work models: A theoretical framework

There is no common conceptualisation about how to understand work models in human service professions. Bunderson [26], however, suggests that a work model can be understood as an integrated unit of practice which allows the replication of information and interaction. It often corresponds to a group of performance tasks. In this study, a work model is understood as a more or less distinct pattern of actions in professional practice. It focuses on how time is spent on various tasks, decision-making processes, and interactions with patients, colleagues and other stakeholders involved in the RTW process.

Moore et al [20] argue that, besides providing a description of the intervention, it is important for studies to look at some key components of how an intervention is implemented. They highlight fidelity (whether the intervention was delivered as intended), dose (the quantity of intervention), and the “reach” of the intervention (whether the intended target group comes into contact with the intervention, and how). These components guided the present study’s mapping of work models. Firstly, the three overall assignments (providing patients with individual support, healthcare collaboration, and external collaboration with other stakeholders in the RTW process) serve as the basis for analysing the work models [23]. Another important factor in how the coordinators shape their work is whether and how they select which patients to work with [24, 25] (i.e. the “reach” of the intervention). Four overall dimensions therefore determine how the work models are defined in this study: selection of patients, individual patient support, healthcare collaboration, and external collaboration. Furthermore, we confine our definition to direct work with patients; we do not include possible additional tasks such as development work of insurance medicine issues or compiling clinics’ sick-leave statistics. Taking fidelity and dose [20] into consideration, we explore how much of the coordinators’ work is related to these four dimensions. Details of the specific chosen work tasks can be found in the methods section and in (see S1 Table).

## Methods

This study was conducted as a cross-sectional study. It included all RTW coordinators in primary healthcare, psychiatry and orthopaedics in one urban region in Sweden. It also uses some questionnaire data collected from patients who had been in contact with the participating coordinators.

### The coordinator dataset

#### Participants and data collection

All RTW coordinators working in primary healthcare, psychiatry and orthopaedics in one region received a questionnaire in February 2020. Three reminders were sent to non-responders in March, April and May 2020. Lists and contact details were provided by contact persons for the coordinator networks in each clinical area.

The study population included 82 coordinators, of whom 73 responded. This gives a response rate of 89% (87.5% in psychiatry and orthopaedics and 89.7% in primary health clinics).

#### The questionnaire

A questionnaire was developed to collect information from RTW coordinators about their work with patient rehabilitation and the RTW process, patient selection, work tasks, work situation, collaboration with stakeholders, educational background, and sociodemographic characteristics. The questionnaire, which contained 41 questions plus partial questions, was developed from previous questionnaires about the work of physicians [27, 28] and coordinators [29] with patients on SA. It was also discussed in a reference group comprising coordinators, physicians and other stakeholders in the RTW process. A pilot study was conducted with ten coordinators from other regions of Sweden. Some amendments were made in accordance with the pilot study.

#### Variables

This study analysed the answers to questions about the coordinators’ experience of patient selection, individual patient support, healthcare collaboration, and external collaboration. A full list of all included questions, response alternatives and the dichotomised or grouped answers used in the analyses are provided as an (see S1 Table). The two questions "How often are you in contact with employers?" and "How often are you in contact with the employment services?" are grouped as one variable, as are the two questions “How often do you attend collaborative meetings with employers?” and “How often do you attend collaborative meetings with the employment services?”.

#### Statistical analysis

The analysis had an explorative approach. The aim was to achieve results for the work model analysis which were as robust as possible for such a complex dataset. Random forest classification was chosen, a machine-learning algorithm that has proved to be an effective tool in prediction and which solves some of the problems of mapping a small but complex dataset at a high level of detail [30]. Random forest classification is an algorithm based on decision trees working as classifiers (see Breiman [30] for a detailed description). Each tree is trained by bootstrapping, using different samples from the data, as well as a random subset of the predicting variables. Many bootstrap samples (here 2500) are selected from the original dataset. Bootstrap samples that are not selected are called out-of-bag observations, and the majority vote of these predict the class (i.e. work model) of an observation. Randomly permuted and modified out-of-bag observations are then passed down the tree to obtain new predictions. The probability of belonging to different classes is estimated by the proportions of out-of-bag predictions in each class [31]. The process of random selection of variables yields out-of-bag error rates (in our case 15.07%, using clinical area as output variable) that are robust with respect to noise. One way to minimize out-of-bag error rates is to look at number of variables to randomly sample as candidates at each split, in this case, three predicting variables at each split were used. Internal estimates monitor the errors, strengths, and correlations, which gives the method the ability to reduce bias and variance and achieve a high overall accuracy rate [30].

Random Forest creates the distance/similarity metrics in unsupervised mode. The actual clustering/classification is made by a more traditional method, Partitioning Around Medoids (PAM). PAM is a good fit for smaller data, while K-means generally is a better fit for bigger data. PAMs strength compared to K-means is that PAM uses medoids (actual points in the dataset) instead of centroids (artificial points). Another measure that was used in the validation process is called Average Silhouette Width (ASW), this validation index is used to optimize the clustering process. Mean Decrease Gini were also used to determine how much each variable contributes to classifying the data compared to the other 18 variables used: the higher the number, the higher the importance of the variable for the classification of the work models.

Descriptive statistics were performed to provide an overall picture of the RTW coordinators’ answers to the selected variables.

### The patient dataset

One variable from a patient dataset used in a previous study was used in this study, see [32] for full details of the patient study.

#### Participants and data collection

Patients in primary healthcare or psychiatry who had been in contact with a RTW coordinator in the particular region, received a questionnaire during the same period as the coordinators received their questionnaire. Of the 292 responding patients (27% response rate), 247 had been in touch with a coordinator who participated in the present study. These were included in this study.

#### Variable and statistical analysis

One variable from the patient dataset (“How often did you have face-to-face meetings with your RTW coordinator?”) was used to examine whether the number of face-to-face meetings reported by the coordinators working according to the different work models corresponds with the number reported by the patients these RTW coordinators had met during the same study period. This was carried out by adding the work model of the patient’s RTW coordinator to the patient dataset. Differences between the identified work models in median face-to-face meetings were tested with Mann Whitney U test.

### Ethical consideration

The study was approved by the Swedish Ethical Review Authority (No. 2020–00403). The first page of the questionnaire contained written information about the study, the legal and ethical requirements for the study, and that the respondents later could withdraw their participation by contacting the principal investigator. The information also contained a statement that respondents provided their informed consent to participate in the study by returning the completed questionnaire to the principal investigator at Karolinska Institutet.

## Results

A large majority of the coordinators were women (90.4%), and only 16.4% were younger than 40 (Table 1). Nearly half of the coordinators were qualified physiotherapists, while 30.1% were occupational therapists, 8.2% were health social workers, and the rest had other vocational backgrounds. Most (63.0%) had worked as coordinators for 1–3 years, and 21.9% more than 3 years. The majority of the coordinators worked in primary healthcare clinics. Most worked part-time as RTW coordinators, with 54.8% working less than 60% of a full-time position. Most combined RTW coordination with other work, often based on their primary profession.

**Table 1:** Characteristics of the RTW coordinators (n = 73), frequencies and percent.

Characteristics	N (%)
Age	
20–39	12 (16.4)
40–54	30 (41.1)
55 years or older	31 (42.5)
Gender	
Women	66 (90.4)
Men	7 (9.6)
Vocational training	
Physiotherapy	34 (46.6)
Occupational therapy	22 (30.1)
Health social work	6 (8.2)
Other*	11 (15.1)
Years as coordinator	
Less than 1 year	11 (15.1)
1–3 years	46 (63.0)
More than 3 years	16 (21.9)
Clinical area	
Primary healthcare clinic	52 (71.2)
Psychiatric clinic	14 (19.2)
Orthopaedic clinic	7 (9.6)
Proportion of work as a coordinator (% of a full-time position)	
Less than 60%	40 (54.8)
60–100%	33 (45.2)

RTW = return to work

* Other healthcare professions, or a mix of training, e.g. in human resources.

### Coordinators’ selection of patients and distribution of work tasks

Table 2 presents the coordinators’ selection of patients and the distribution of work tasks regarding individual patient support, healthcare collaboration, and external collaboration. Most did not have particular criteria for selecting patients (and thus dealt with all patients who wanted support in their RTW process), and did not refuse cases that other staff referred to them (84.9%). Nearly half (46.6%) stated that they were not supposed to deal with patients who have been on SA for more than 180 days. A minority answered that they did not contact employers (29.6%) or SIA officers (35.3%) but rather let the patients deal with those contacts themselves. A larger proportion of the respondents spent more than half of their time on telephone contacts (27.4%) compared to face-to-face meetings (17.8%). A majority (56.1%) were in contact with employers every week and attended collaboration meetings with employers at least a few times a month (65.8%). A clear majority never or only occasionally attended collaboration meetings with SIA, the employment services, or social services. Nearly all (91.8%), however, were in touch with the SIA at least a few times a month.

**Table 2:** RTW coordinators’ (n = 73) answers to questions about: selection of patients, individual patient support, healthcare collaboration, and external collaboration, frequencies and percentages.

Questionnaire items/statements	Response alternative	N (%)
***Selection of patients***		
Do you have criteria for which patients you select to work with as a coordinator?		
…patients at risk of being on SA	Yes	29 (39.7)
	No	44 (60.3)
…patients who have been denied SA	Yes	15 (20.5)
	No	58 (79.5)
…I only take on cases when I think my measures can promote faster RTW	Yes	20 (27.4)
	No	53 (72.6)
I refuse patients referred to me by other staff	Yes	11 (15.1)
	No	62 (84.9)
I am not supposed to deal with patients who have been on SA for more than 180 days	Agree	34 (46.6)
	Do not agree	38 (52.1)
***Individual patient support***		
How often do you talk to patients about the national sickness certification guidelines?	Often	43 (58.9)
	Seldom	30 (41.0)
I don’t contact the employers, I let the patients deal with that contact	Agree	21 (29.6)
	Do not agree	50 (70.4)
I don’t contact SIA officers, I let the patients deal with that contact	Agree	25 (35.3)
	Do not agree	46 (64.7)
Proportion of time spent on the following:		
…telephone contacts*	50% or more	20 (27.4)
	Less than 50%	53 (72.6)
…face-to-face meetings with patients*	50% or more	13 (17.8)
	Less than 50%	60 (82.2)
***Healthcare collaboration***		
I usually give physicians suggestions about how patients’ RTW can be promoted	Yes	59 (82.0)
	No	13 (18.1)
How often are you in contact with patients’ healthcare providers outside your unit?	Every week	13 (17.8)
	A few times a month	28 (38.4)
	A few times a year/Never	32 (43.8)
How often are you in contact with employers?	Every week	41 (56.1)
	A few times a month	24 (32.9)
	A few times a year/Never	8 (10.9)
How often are you in contact with the employment services?	Every week	11 (15.0)
	A few times a month	21 (28.8)
	A few times a year/Never	41 (56.2)
How often do you attend collaboration meetings with employers?	Every week	20 (27.4)
	A few times a month	28 (38.4)
	A few times a year/Never	25 (34.2)
How often do you attend collaboration meetings with the employment services?	Every week	6 (8.2)
	A few times a month	11 (15.1)
	A few times a year/Never	56 (76.7)
How often do you make workplace visits?	Every week	10 (13.7)
	A few times a month	22 (30.1)
	A few times a year/Never	41 (56.2)
How often are you in contact with the SIA?	Every week	36 (49.3)
	A few times a month	31 (42.5)
	A few times a year/Never	6 (8.2)
How often do you participate in collaboration meetings with the SIA?	Every week	10 (13.7)
	A few times a month	16 (21.9)
	A few times a year/Never	47 (64.4)
How often are you in contact with the social services?	Every week	11 (15.1)
	A few times a month	9 (12.3)
	A few times a year/Never	53 (72.6)
An important task for coordinators is to encourage patients’ and employers’ collaboration regarding RTW	Agree	67 (91.8)
	Do not agree	5 (6.7)

SA = sickness absence; RTW = return to work; SIA = Social Insurance Agency

* See S1 Table for details

### Mapping the coordinators’ work models

Three work models were identified in the Random forest classification tree analysis. Model A was the most common, used by 54 coordinators (74%), while model B was used by 12 (16%) and model C seven coordinators (10%). In the following, we will present how the work models differ in relation to the four identified dimensions: patient selection, individual patient support, healthcare collaboration and external collaboration.

### Selection of patients

**Fig 1:**
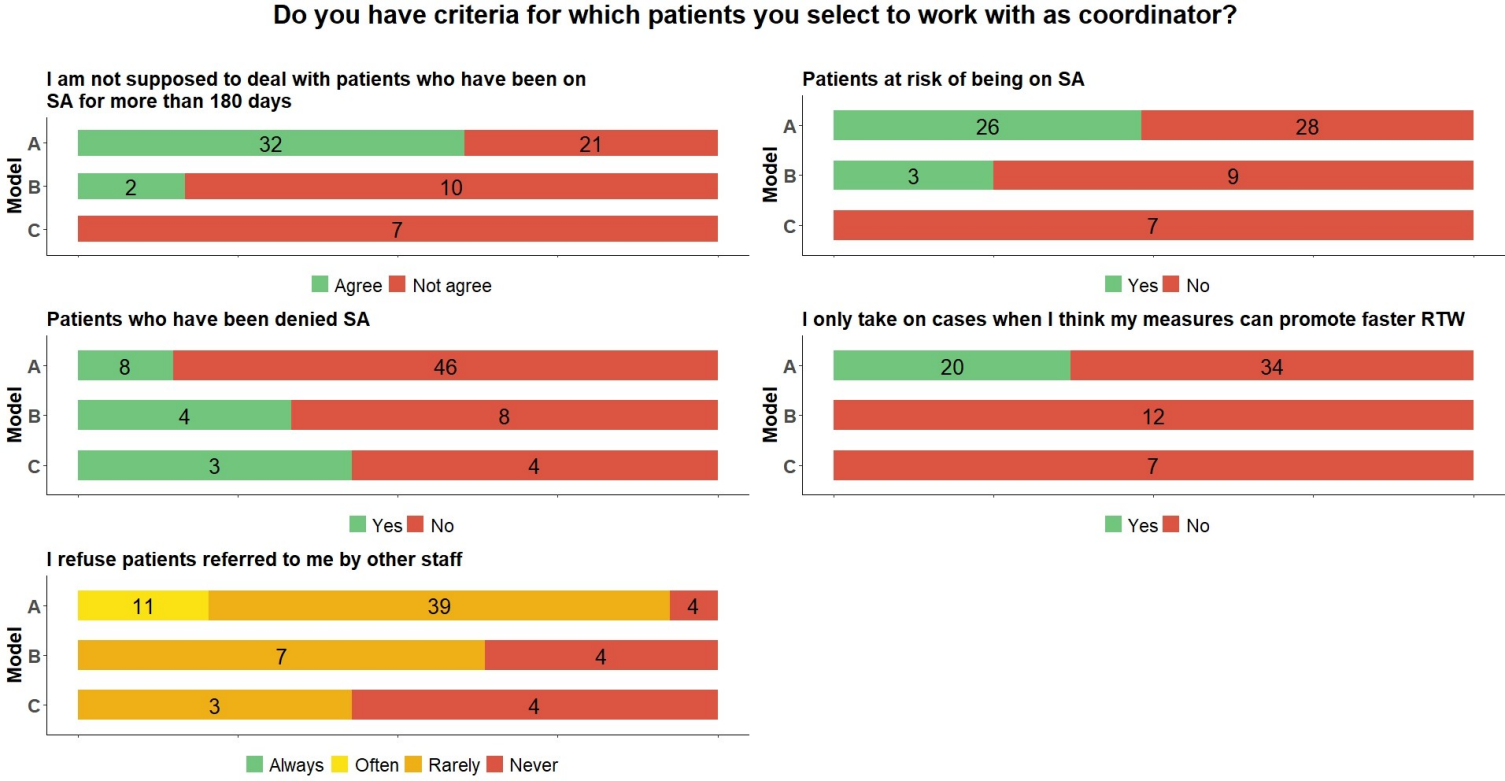
Distribution of RTW coordinators’ (N = 73) answers to questions about the selection of patients across the three models (frequencies). SA = sickness absence; RTW = return to work.

As Fig 1 shows, model A is characterised by a higher number of coordinators who did not take on patients who had been on SA for more than 180 days or who had been denied SA. Almost half of the coordinators in model A chose actively to work with patients at risk of SA, while this was not an active choice for those in model C.

All those who answered that they only take on SA cases when they believe their measures can promote faster RTW were found in model A. A clear majority in model A rarely refused patients referred to them by other professionals. At the same time, all those who responded that they often refuse patients, were also found in model A. About half of those in model B rarely refused patients, while the majority of those in model C said that they never did so.

### Individual patient support

**Fig 2:**
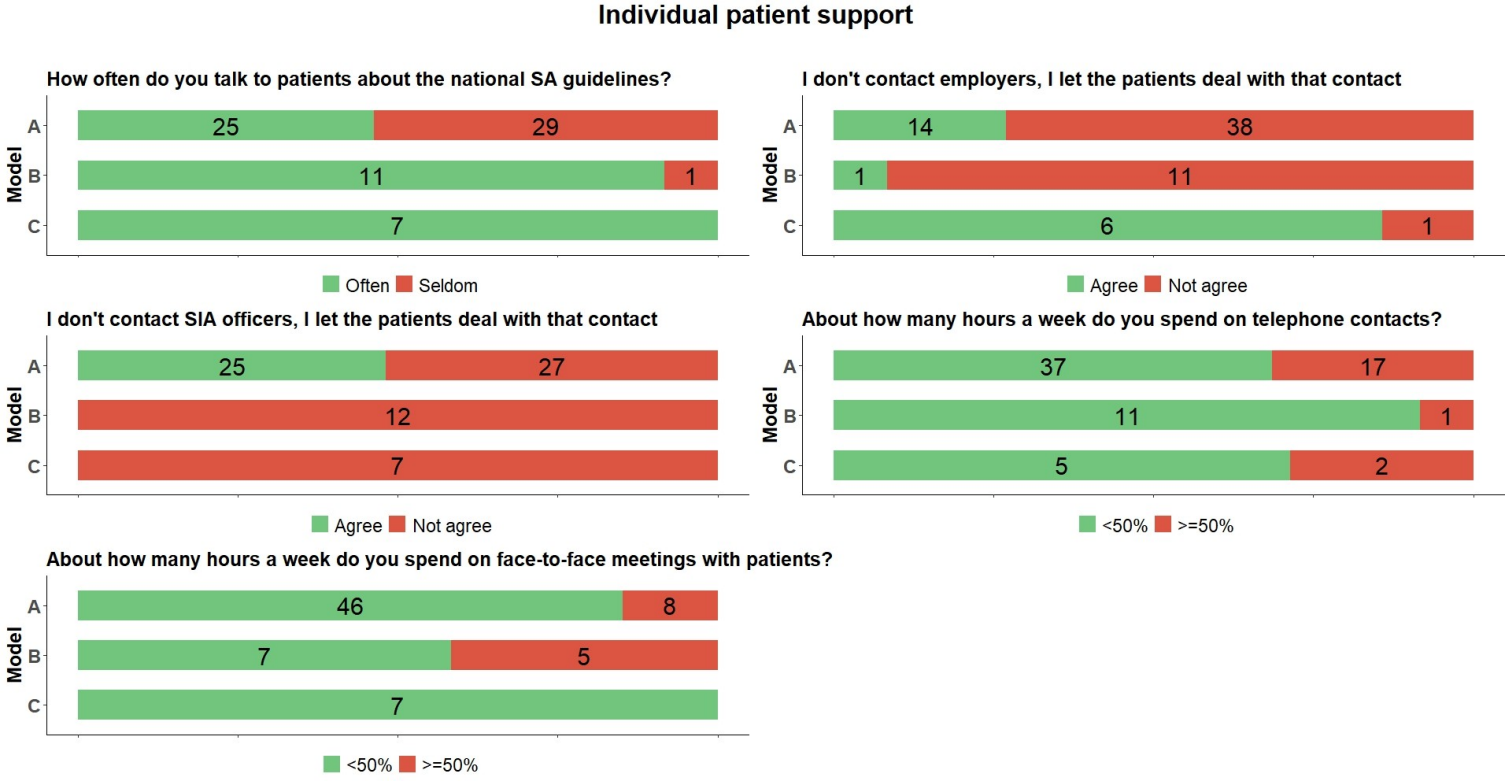
Distribution of RTW coordinators’ (N = 73) answers to questions about individual patient support across the three models (frequencies). SA = sickness absence; SIA = Social Insurance Agency.

All coordinators in model C talked to patients about the national SA guidelines, while less than half in model A did so (Fig 2). Almost all in model C said that they let the patients themselves deal with contacts with their employer. This was less common in model A and even less so in model B. In model A, almost half answered that they let patients themselves deal with contacts with their SIA officer, while none in model B and C did so.

We also looked at the number of hours (weighted based on working hours) coordinators spent on telephone respectively face-to-face meetings with patients. We found that model B had the highest proportion of face-to-face meetings, while coordinators in models A and C spent more time in telephone meetings.

In a dataset with questionnaire responses from patients who had recently been in contact with a RTW coordinator in the present study, 221 patients had met a coordinator working according to model A and 26 patients had met a coordinator working according to model B. No patients in the sample had met a coordinator working according to model C. Patients who had been in touch with a coordinator working according to model B reported a median of 2 (interquartile range 1–4) face-to-face meetings with the coordinator, while those who had met a coordinator working according to model B reported a median of 4 (interquartile range 2–6) face-to-face meetings. A Mann Whitney U test showed that there was a statistically significant difference between models A and B in the number of face-to-face meetings (U = 1777.50, p = .002).

### Healthcare collaboration

**Fig 3:**
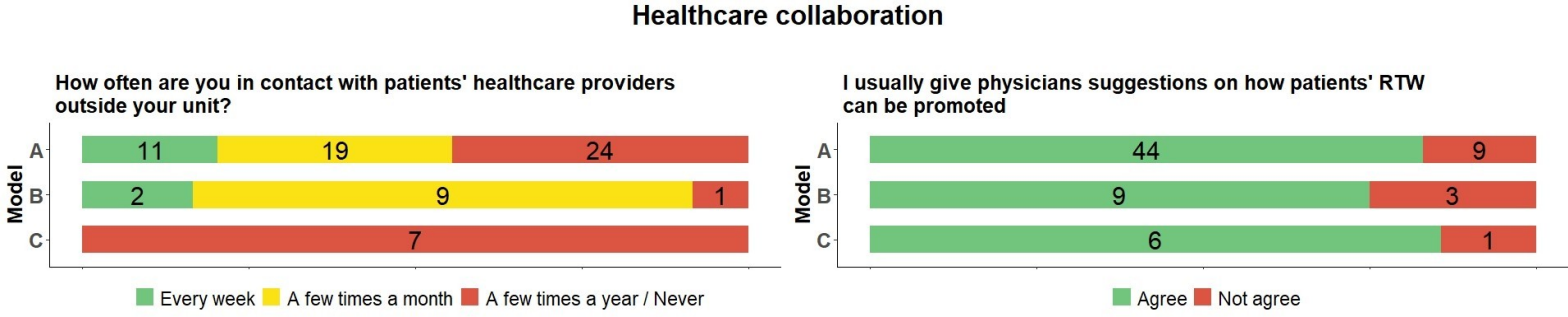
Distribution of RTW coordinators (N = 73) answers to questions about healthcare collaboration across the three models (frequencies). RTW = return to work.

There were major differences in how often coordinators were in contact with patients’ healthcare contacts outside their own unit (Fig 3). In model B, a clear majority had such contacts every month or week, compared to slightly more than half in model A. In model C, this type of contact happened just a few times a year or never. There were, however, no major differences between coordinators regarding whether they usually gave physicians suggestions about how patients’ RTW can be promoted: a majority in all models stated that they did so.

### External collaboration

**Fig 4:**
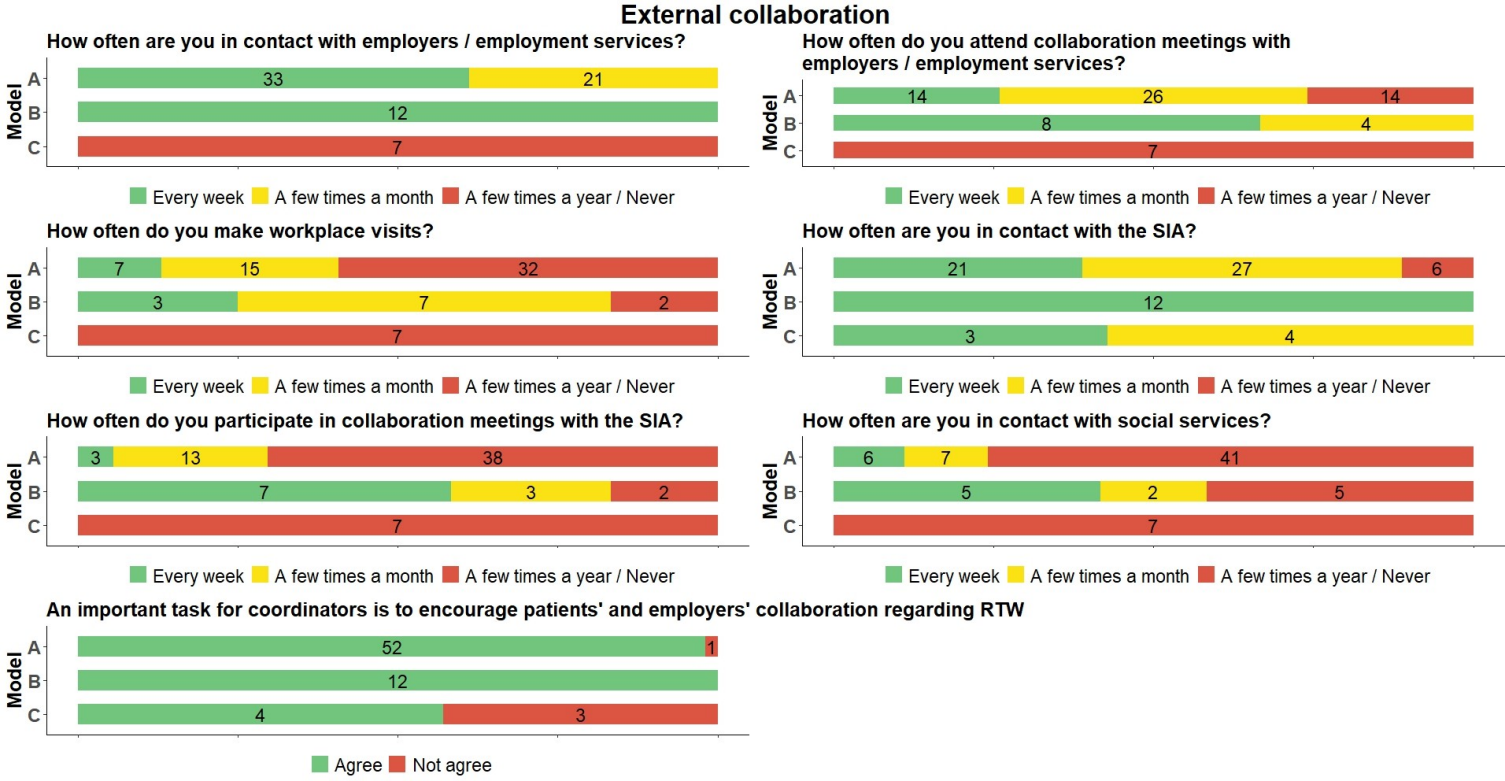
Distribution of RTW coordinators’ (N = 73) answers to questions about external collaboration across the three models (frequencies). SIA = Social Insurance Agency; RTW = return to work.

Regarding external collaboration, model C stands out significantly because these coordinators were never–or only a few times per year–in contact with other stakeholders. The exception was the SIA, with whom all were in contact with at least once a month (Fig 4). There were also differences between models A and B. Coordinators in model B had many more contacts and meetings with employers/employment services, the SIA, and the social services than those in model A. A clear majority in model B also visited patients’ workplaces, while less than half of the coordinators in model A did so.

### Characteristics of the three work models

As the above results show, the differences between the work models are about the degree to which they perform certain work tasks, whom they collaborate with, or to what extent they select certain groups of patients to work with.

**In work model A**, coordinators were most likely to select their patients. These patients were less likely to be on long-term SA or those who had been denied SA, but more likely to be at risk of SA. That to say, the coordinators actively chose to work early in the RTW process. Furthermore, they are more likely both to take on SA cases when they believe their measures can promote faster RTW and to refuse patients referred to them by other professionals. Regarding the questions of individual support to patients, less than half talked to patients about the national SA guidelines. A minority answered that they let the patients themselves deal with contacts with their employer and almost half said that they let the patients themselves deal with contacts with their SIA officer. A small group spent 50% or more of their time in face-to-face meetings with patients, while twice that number (about a third) spent 50% or more of their time on telephone contacts. Regarding healthcare collaboration, slightly more than half were in contact with patients’ healthcare contacts outside their own unit every month or week. Similar results were found for external collaboration. All were in contact with employers/employment services at least every month, with a third every week. Two thirds also participated in collaborative meetings with employers at least every month, and more than a third visited their patients’ workplaces. Nearly all had contact with the SIA at least every month, with a third every week. Less than a third participated in collaborative meetings with the SIA, and a quarter were in contact with the social services at least once a month.

**In work model B**, fewer coordinators actively selected patients at risk of SA or who had been denied SA. Slightly more than half rarely refused patients referred to them by other professionals, while the rest seldom or never did so. In their individual support to patients, nearly all coordinators talked to patients about the national SA guidelines. No one in model B said that they let patients themselves deal with contacts with their SIA officer, while nearly none left their patients to deal with contacts with their employer. Model B is characterised by a higher proportion of face-to-face meetings with patients (nearly half spent 50% or more of their time in face-to-face meetings), and very few spent 50% or more of their work time on telephone contacts.

What clearly characterises model B is the large amount of both healthcare and external collaboration. A clear majority were in contact with patients’ healthcare contacts outside their own unit every month or week. Regarding collaboration with external stakeholders, all were in contact with SIA and employers/employment services every week, and almost all participated in collaborative meetings with employers and SIA at least every month. A clear majority in model B also visited the patients’ workplaces, and more than half had contact with social services at least once a month.

**In work model C**, coordinators generally did not actively select certain groups of patients to work with, and the few who did so focused on patients who had been denied SA. Nor did these coordinators refuse patients referred to them by other professionals. In their work with individual support to patients, all coordinators talked to patients about the national SA guidelines, and almost all said that they let the patients deal with their own contacts with their employer. None, however, left patients to deal with contacts with their SIA officer. None spent 50% or more of their work time in face-to-face meetings with patients, and a minority spent that much on telephone contacts.

What clearly characterises model C is the low extent of both healthcare and external collaboration. These coordinators all answered that they were never, or only a few times a year, in touch with patients’ healthcare contacts outside their own unit. Similarly, they never or only a few times a year had contact with or participated in meetings with other stakeholders. The exception to this was the SIA, with whom they were all in contact at least every month.

### The distribution of the work models across clinical areas

**Fig 5:**
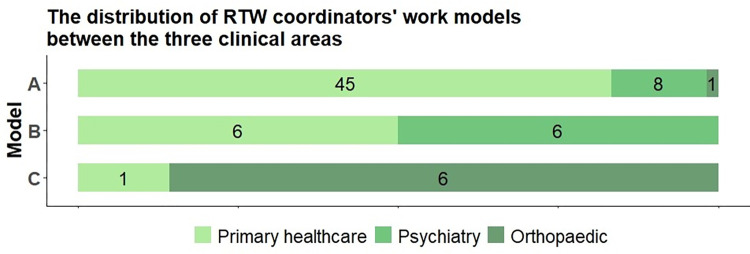
The distribution of RTW coordinators’ work models across the three clinical areas.

The work models were distributed differently across the three clinical areas. As Fig 5 shows, model A was more common in primary healthcare clinics, and model C was more common in orthopaedic clinics, while model B was distributed equally between primary healthcare and psychiatric clinics.

### Variable importance for model classification

**Fig 6:**
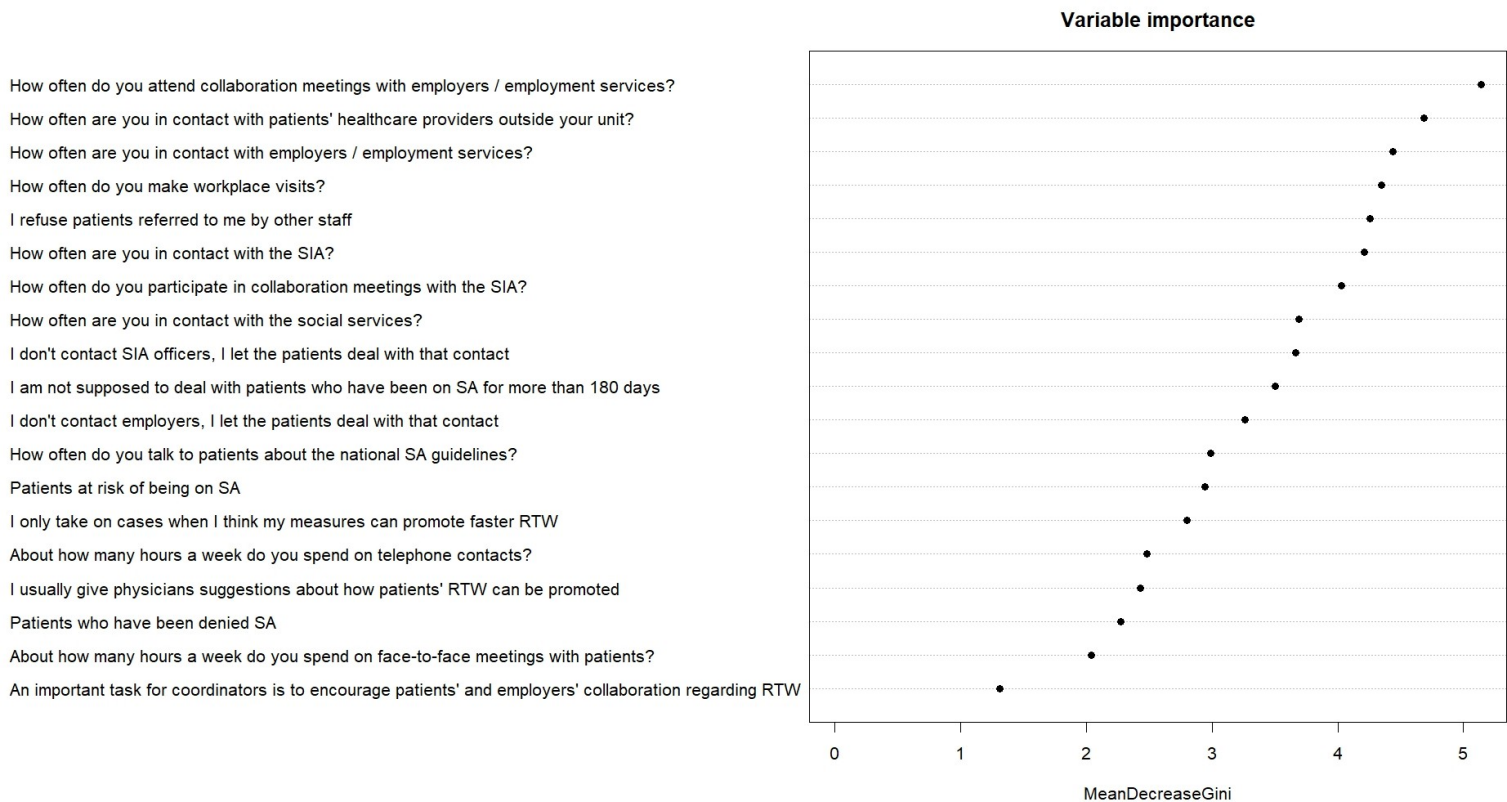
The variables’ importance for determining the work models, falling order. SIA = Social Insurance Agency; SA = sickness absence; RTW = return to work.

Fig 6 shows which variables were most important in identifying the three models. Attending meetings with employers or the employment services was the most important variable for classification, while believing that it is important for coordinators to encourage patient/employer collaboration around RTW, was of least importance.

## Discussion

In this study, we have explored the work models used by RTW coordinators in three different clinical settings: primary healthcare, psychiatry and orthopaedics. With the aim of mapping and describing the different work models, we explored how often or how much time the coordinators spent on certain tasks, how much they collaborated with various stakeholders, and whether they actively selected certain groups of patients. We identified three models which all have different approaches to the coordinators’ central assignments of giving individual support to persons on SA, collaborating within healthcare, and collaborating externally with other stakeholders involved in the RTW process. Work model B seems to be the model that corresponds most to these assignments, as it displays the greatest amount of collaboration with both healthcare and external contacts. Work model B also involved most face-to-face meetings with patients (also confirmed by responses to a patient questionnaire), which the systematic review by Dol et al. [19] found strong evidence for increasing RTW rates. Work model B seems to have what Moore et al. [20] call higher fidelity (i.e. the coordination measures were delivered as intended) and higher dose (i.e. a higher number of coordination measures). Work model C, on the other hand, corresponds very little to the expectations about collaboration with healthcare and external stakeholders, which indicates low fidelity as well as low dose. Models B and C thus displayed clearly different patterns of actions, thus mirroring different integrated units of practices among coordinators in the respective work model [26]. Coordinators working according to model A chose a middle-way with regard to the assignment of collaboration with healthcare and external stakeholders, but achieved less “reach” (i.e., not all in the target group of people on SA were offered coordination) [20]. This study cannot answer why RTW coordinators actively deselect for example people who have exceeded 180 SA days, or if these people receive any support with RTW coordination from other professionals. Taking Moore et al’s [20] perspective on the importance of evaluating the reach of an intervention, it is important to discuss whether, and if so how, deselected people receive qualified RTW coordination, and to improve the conditions and possibilities for RTW coordinators to achieve better reach.

A previous study [15] found that coordinators in primary healthcare felt they lacked a clear description of how to implement their role into their daily work practice. This led to a variation in their practices and the risk of drift from their original assignments. These primary healthcare coordinators did, however, have a network in which they discussed how to interpret their role and their assignments and shared best practice. Such coordinator networks also exist for the participants in the present study, one for each clinical area. This can lead to what Bunderson [26] calls integrated units of practice, communicated and replicated by coordinators in the networks. This could be one explanation of the tendency for the use of the different work models to vary across the clinical areas.

The above finding highlights some overall differences between primary healthcare, psychiatry and orthopaedics with regard to how coordinators carry out their work. The clinical contexts, with their different patient populations, have an important influence on how work models are shaped. Work model C was clearly dominated by coordinators in orthopaedic clinics, who have apparently developed this model on the basis of other grounds and arguments than the assignment to collaborate with healthcare and the different external stakeholders. In this study, we have not explored the reasons behind applying different work models–this is a question best answered by interviewing RTW coordinators. One explanation could be different interpretations of the concept of coordinating, with some interpreting it as actively making contact and collaborating with stakeholders, while others may interpret coordination as encouraging others to get in touch and collaborate with each other. The fact that the average age of the coordinators is high indicates that they have long experience of working in the professions for which they originally trained. As reported previously [10, 11, 15], the content of RTW coordination is not clearly described in the Swedish context. Coordinators may therefore apply different work models on the basis of expertise and experience gained from their respective base professions. Other reasons could be differences in patient needs and resources, local rehabilitation policies, available resources in relation to workload, organisational culture and leadership at local level. This would result in different outcomes of RTW coordinators’ practices and applied work models. While previous international review studies stress the importance of better communication and collaboration around people on SA in their RTW process [1, 2], especially between employers and healthcare providers [3, 33], these and other studies about RTW coordination seldom give an in-depth understanding of what kind of collaboration is needed to achieve positive results. As pointed out previously, collaboration can be time-consuming [8]; time constraints and various organisational demands in different settings and countries may oblige some RTW coordinators to do less collaboration. In the end, ideal working practices are often constrained by the realities of clinical practice–realities that affect to what extent RTW coordinators are able to combat insufficient RTW collaboration and the high rates of SA. However, with the work models outlined, it is possible to evaluate them from different perspectives. For example, it will be possible to compare the model’s association with the length of SA among patients, and how they are perceived by the patients, the physicians and other colleagues the RTW coordinators collaborate with.

### Strengths and limitations

Although this study is based on a relatively small sample, the high response rate of 89% means that the results probably would not change considerably if the nine non-responding RTW coordinators also had participated. We have no background information about those who did not respond, and no indication that those who did not participate worked in a significantly different way or had a higher workload than those who responded. It can therefore be assumed that there is a reasonable degree of generalisation of the findings for the RTW coordinators in the studied region.

It is possible that coordinators in other clinical areas or regions differ to some extent with regard to the variety of vocational trainings, which may lead to that they work differently. If this is the case, including data from several regions could have resulted in additional or different work models. One should be careful when attempting to generalize these findings to RTW coordinators in other countries, as they often work in other contexts, such as at insurance companies or workplace organisations [18].

One limitation of this study is that it has not included the full range of work tasks. We chose to focus on those directly linked to the main assignments for RTW coordinators in Sweden, and excluded other possible tasks such as development work of insurance medicine issues or compiling clinics’ sick-leave statistics. By using Mean Decrease Gini we were, however, able to determine how much each included variable/task contributed in classifying the data, that is to say, which variables were most important in identifying differences between the three different models. Mean Decrease Gini showed (see Fig 6) that collaboration meetings with employer/employment services was the most important variable in identifying differences. Face-to-face meetings had the second lowest importance. However, by using some data collected from the RTW coordinators’ patients we were able to confirm that the median amount of face-to-face meetings differed significantly between models A and B, and therefore was a variable that distinguish these models. This indicates that most (if not all) of the included variables were important for the Random Forrest Classification analysis, that is to say, all variables seem to have contributed in classifying the data.

Ideally, a combination of quantitative and qualitative methods should be used to fully understand complex interventions [20]. The lack of qualitative data in this study means that it is not possible to describe the content of the support provided by the coordinators. It is therefore not possible to draw any conclusions about possible differences between the models regarding what specific issues RTW coordinators focus on, or the quality of their interventions. Further, it has not been possible to explore why RTW coordinators chose to work with the different models. This is an important subject for future research.

## Conclusion

This cross-sectional study is the first to explore differences in RTW coordination practices. We identified three work models. The differences between the models lie in how much they actively select certain patients, how much they collaborate, and with whom. The models thus differ in how well they correspond to the coordinators’ assignments to give individual support, collaborate in healthcare, and collaborate externally with other stakeholders involved in the RTW process. The fact that these three models are distributed differently across primary healthcare, psychiatry and orthopaedics, indicates that the clinical context and its organisational prerequisites have an important influence on how work models are shaped in practice.

## Supporting information

S1 Table (DOCX)
