# Supplementary material for: Coordinators in the return-to-work process: Mapping their work models
Source: PLoS One. 2023 Aug 10;18(8):e0290021. doi: 10.1371/journal.pone.0290021 (PMC10414594; doi:10.1371/journal.pone.0290021)
Supplement: S1 Table — (DOCX) [file pone.0290021.s001.docx]

**S1 Table. Appendix.** A list of all included questions, response alternatives and the dichotomised or grouped answers used in the Random Forest Classification analyses.

|  | **Response alternatives and if/how they were dichotomised/grouped** | |
| --- | --- | --- |
| **Complete question** | **Response alternatives** | **Dichotomisation/groups used** |
| ***Selection of patients*** |  |  |
| Do you have criteria for which patients you select to work with as a coordinator? |  |  |
| …patients at risk of being on SA | Yes, No | *No changes* |
| …patients who have been  denied SA | Yes, No | *No changes* |
| …I only take on cases when I think my measures can promote faster RTW | Yes, No | *No changes* |
| I refuse patients referred to me by other staff | Always, Often, Rarely, Never, Not applicable | *No changes* |
| I am not supposed to deal with patients who have been on SA for more than 180 days | Totally agree, Agree to large extent, Hardly agree, Do not agree at all | Agree (Totally agree/Agree to large extent) vs  Not agree (Hardly agree/Do not agree at all) |
| ***Individual patient support*** |  |  |
| How often do you talk to patients about the national SA guidelines? | Always, Often, Quite often, Quite seldom, Seldom, Never | Often (Always, Often, Quite often) vs  Seldom (Quite seldom, Seldom, Never) |
| I don’t contact employers, I let the patients deal with that contact | Totally agree, Agree to large extent, Hardly agree, Do not agree at all | Agree (Totally agree/Agree to large extent) vs  Not agree (Hardly agree/Do not agree at all) |
| I don’t contact SIA officers, I let the patients deal with that contact | Totally agree, Agree to large extent, Hardly agree, Do not agree at all | Agree (Totally agree/Agree to large extent) vs  Not agree (Hardly agree/Do not agree at all) |
| About how many hours a week do you spend on the following tasks? |  |  |
| …telephone contacts | Free text answer | Number of hours is weighted based on total working hours a week as coordinator. Grouped as:  50% or more of work time  Less than 50% of work time |
| …face-to-face meetings  with patients | Free text answer | Number of hours is weighted based on total working hours a week as coordinator. Grouped as:  50% or more of work time  Less than 50% of work time |
| ***Healthcare collaboration*** |  |  |
| How often are you in contact with patients' healthcare providers outside your unit? | More than 10 times a week, 6-10 times a week, 1-5 times a week, A few times a month, A few times a year, Never/almost never | Answer 1, 2 or 3 = "Every week"  Answer 4 = "A few times a month"  Answer 5 or 6 = "A few times a year/Never" |
| I usually give physicians suggestions about how patients’ RTW can be promoted | Totally agree, Agree to large extent, Hardly agree, Do not agree at all | Agree (Totally agree/agree to large extent) vs  Not agree (Hardly agree/Do not agree at all) |
| ***External collaboration*** |  |  |
| How often are you in contact with employers? | More than 10 times a week, 6-10 times a week, 1-5 times a week, A few times a month, A few times a year, Never/almost never | Answer 1, 2 or 3 = "Every week"  Answer 4 = "A few times a month"  Answer 5 or 6 = "A few times a year/Never" |
| How often are you in contact with the employment services? | More than 10 times a week, 6-10 times a week, 1-5 times a week, A few times a month, A few times a year, Never/almost never | Answer 1, 2 or 3 = "Every week"  Answer 4 = "A few times a month"  Answer 5 or 6 = "A few times a year/Never" |
| How often do you attend collaboration meetings with employers? | More than 10 times a week, 6-10 times a week, 1-5 times a week, A few times a month, A few times a year, Never/almost never | Answer 1, 2 or 3 = "Every week"  Answer 4 = "A few times a month"  Answer 5 or 6 = "A few times a year/Never" |
| How often do you attend collaboration meetings with the employment services? | More than 10 times a week, 6-10 times a week, 1-5 times a week, A few times a month, A few times a year, Never/almost never | Answer 1, 2 or 3 = "Every week"  Answer 4 = "A few times a month"  Answer 5 or 6 = "A few times a year/Never" |
| How often do you make workplace visits? | More than 10 times a week, 6-10 times a week, 1-5 times a week, A few times a month, A few times a year, Never/almost never | Answer 1, 2 or 3 = "Every week"  Answer 4 = "A few times a month"  Answer 5 or 6 = "A few times a year/Never" |
| How often are you in contact the SIA? | More than 10 times a week, 6-10 times a week, 1-5 times a week, A few times a month, A few times a year, Never/almost never | Answer 1, 2 or 3 = "Every week"  Answer 4 = "A few times a month"  Answer 5 or 6 = "A few times a year/Never" |
| How often do you participate in collaboration meetings with the SIA? | More than 10 times a week, 6-10 times a week, 1-5 times a week, A few times a month, A few times a year, Never/almost never | Answer 1, 2 or 3 = "Every week"  Answer 4 = "A few times a month"  Answer 5 or 6 = "A few times a year/Never" |
| How often are you in contact with the social services? | More than 10 times a week, 6-10 times a week, 1-5 times a week, A few times a month, A few times a year, Never/almost never | Answer 1, 2 or 3 = "Every week"  Answer 4 = "A few times a month"  Answer 5 or 6 = "A few times a year/Never" |
| An important task for coordinators is to encourage patients’ and employers’ collaboration regarding RTW | Totally agree, Agree to large extent, Hardly agree, Do not agree at all | Agree (Totally agree/agree to large extent) vs  Not agree (Hardly agree/Do not agree at all) |

SA = sickness absence; RTW = return to work; SIA = Social Insurance Agency
